# Generation and characterization of ErbB2-CAR-engineered cytokine-induced killer cells for the treatment of high-risk soft tissue sarcoma in children

**DOI:** 10.18632/oncotarget.19821

**Published:** 2017-08-02

**Authors:** Michael Merker, Verena Pfirrmann, Sarah Oelsner, Simone Fulda, Thomas Klingebiel, Winfried S. Wels, Peter Bader, Eva Rettinger

**Affiliations:** ^1^ Division for Stem Cell Transplantation and Immunology, Department for Children and Adolescents, University Hospital Frankfurt, Goethe University, Frankfurt, Germany; ^2^ Institute for Tumor Biology and Experimental Therapy, Georg-Speyer-Haus, Frankfurt, Germany; ^3^ Institute for Experimental Cancer Research in Pediatrics, Goethe University, Frankfurt, Germany; ^4^ German Cancer Consortium (DKTK), Heidelberg, Germany; ^5^ German Cancer Research Center (DKFZ), Heidelberg, Germany

**Keywords:** soft tissue sarcoma, cell therapy, cytokine-induced killer cells, chimeric antigen receptor, pediatric cancer

## Abstract

Pediatric patients with recurrent, refractory or advanced soft tissue sarcoma (STS) who are simultaneously showing signs of cumulative treatment toxicity are in need of novel therapies. In this preclinical analysis, we identified ErbB2 as a targetable antigen on STS cells and used cytokine-induced killer (CIK) cells transduced with the lentiviral 2^nd^-generation chimeric antigen receptor (CAR) vector pS-5.28.z-IEW to target ErbB2-positive tumors. Solely CIK cell subsets with the CD3^+^ T cell phenotype showed up to 85% cell surface expression of the respective CAR. A comparison of wildtype (WT), mock-vector and ErbB2-CAR-CIK cells showed, that engineered cells exhibited diminished *in vitro* expansion, retained WT CIK cell phenotype with higher percentages of differentiated effector memory/effector cells. Activating natural killer (NK) cell receptor NKG2D-restricted target cell recognition and killing of WT and ErbB2-CAR-CIK cells was maintained against ErbB2-negative tumors, while ErbB2-CAR-CIK cells demonstrated significantly increased cytotoxicity against ErbB2-positive targets, including primary tumors. ErbB2-CAR- but not WT CIK cells proliferated, infiltrated and efficiently lysed tumor cell monolayers as well as 3D tumor spheroids.

Here, we demonstrate a potential cell therapeutic approach using ErbB2-CAR-CIK cells for the recognition and elimination of tumor cells expressing ErbB2, which we identified as a targetable antigen on high-risk STS cells.

## INTRODUCTION

Pediatric patients with very high-risk soft tissue sarcoma (STS) often acquire refractory disease or experience relapse and are simultaneously particularly susceptible to chemotherapy- and radiotherapy-related adverse effects because of cumulative treatment toxicity. Specifically, metastatic alveolar rhabdomyosarcoma (aRMS or RMA) in patients above the age of 10, as well as refractory or relapsed rhabdomyosarcoma (RMS), may not be cured by conventional therapy.

As an experimental approach, allogeneic stem cell transplantation and subsequent immunotherapy may be considered in such patients, with the aim of establishing a new immune system and further augmenting the graft-versus-tumor immune response post-transplant. Easy and manageable *ex vivo* expansion in accordance with good manufacturing practices (GMP) [1-3], wide non-major histocompatibility complex (MHC)-restricted cancer cell recognition and killing as well as low alloreactive activity in preclinical [4, 5] and clinical studies [6, 7], are features of cytokine-induced killer (CIK) cells suggestive of their promise as immune effectors for innovative immune therapeutic interventions in patients transplanted for relapsed or refractory STS. But, in our previous study, even though disease recurrence was delayed or even prevented after allogeneic stem cell transplantation and allogeneic CIK cell interventions, the outcome in our cohort was dismal due to the occurrence of relapse and treatment-related complications (manuscript in preparation). However, the great promise of any kind of cancer immunotherapy still is to clear the tumor without providing additional toxicity.

In this context, chimeric antigen receptor (CAR)-engineered immune cells redirected to recognize tumor-specific antigens are currently under investigation in preclinical and clinical studies. ErbB2 (HER2/neu), a member of the epidermal growth factor (EGF) receptor tyrosine kinase family, is often (over)-expressed in breast cancer and other malignancies, such as brain tumors and sarcomas, but not on hematopoietic cells and may therefore represent an appropriate tumor antigen for targeted immune therapies [8].

The use of CAR-engineering strategies has thus far been mainly confined to a pure T lymphocytes population [9-15]. Hence, little is known about the possibility of CAR-engineering of a heterogeneous immune effector cell population, such as CIK cells, which comprise of T cells, natural killer (NK) cells, and T-NK cells. However, promising preclinical results have recently been reported by studies using CAR-engineered CIK cells [16] against CD19 [17, 18] or CD33/CD123 leukemia targets [19, 20]. Therefore, we hypothesize, that adding tumor antigen-specificity, such as ErbB2-CAR-specificity, to CIK cells that are already capable of NK cell antitumor activity may result in more precise tumor recognition and enhanced cytotoxicity against STS tumors expressing the ErbB2 antigen, such as RMS, thereby providing minimal toxicity risk.

Here, we report preclinical data on ErbB2 as a targetable antigen on high-risk RMS. Several tumor models are established and used for functional analysis. We also confirm that ErbB2-engineered CIK cells, unlike wildtype (WT) CIK cells, are highly active immune effectors with respect to the recognition and clearance of ErbB2-expressing tumors, a finding supportive of the feasibility and efficacy of this potential treatment approach [21].

## RESULTS

### Generation and expansion of ErbB2-CAR CIK cells

**Figure 1:**
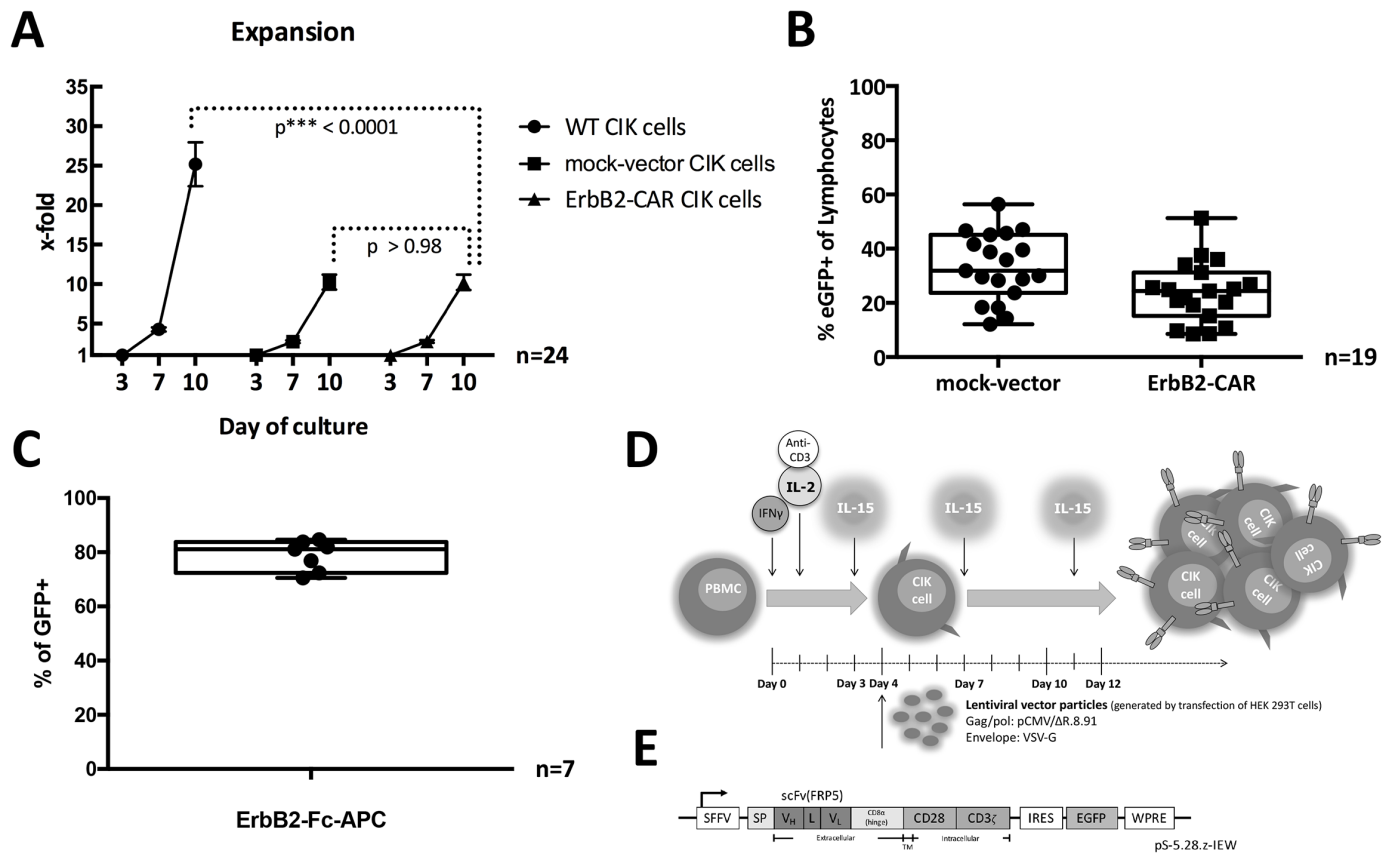
**(A)** Expansion. Expansion rates of WT, mock-vector, and ErbB2-CAR CIK cells on days 3, 7, and 10 of culture (all mononuclear cells were counted) are shown. Proliferation of ErbB2-CAR and mock-vector CIK cells was adequate, but was diminished in the presence of viral vector, which was added on day 4 or 5 of culture, compared with WT CIK cells (p < 0.0001). Expansion rates were not significantly different between mock-vector and ErbB2-CAR CIK cells (p > 0.98). These findings likely relate to non-specific toxicity of the vector itself. **(B)** Transduction rate. Percentage of mock-vector-transduced (mean 33.25% eGFP-positive cells of all gated lymphocytes, SD ± 12.4, range 12.1 – 56.4, n = 19 s) and ErbB2-CAR vector-transduced (mean 23.78% eGFP-positive cells of all gated lymphocytes, SD ± 11.1, range 8.5 – 51.3%, n = 19) CIK cells are shown by flow cytometry after 12 days of *in vitro* culture. Transduction rates were not significantly different between mock-vector- and ErbB2-CAR vector-transduced CIK cells. Transduction was donor-dependent, but was feasible in all cases. **(C)** CAR surface expression. The surface expressions of the ErbB2-specific CAR on ErbB2-CAR vector-transduced cells after 12 days of culture are shown by flow cytometry (mean 78.8% CAR surface expression of all eGFP-positive (=transduced) cells, SD ± 5.7, range 70.6 - 84.6%, n = 7). **(D)** Generation of ErbB2-CAR CIK cells. CIK cells were generated from PBMCs by stimulation with IL-2, IFN-γ and monoclonal anti-CD3 antibody on day 0 and 1 as well as by stimulation with IL-15 every 3 to 4 days over a culture period of 12 days. CAR engineering was performed on day 4 or 5 using VSV-G pseudotyped lentiviral vector particles generated by transfection of HEK 293T cells. **(E)** CAR sequence. The CAR sequence consists of an IgG heavy-chain signal peptide, an ErbB2-specific antibody fragment scFv (FRP5) and a modified CD8α hinge region, as well as CD28 transmembrane and intracellular domains and a CD3ζ intracellular domain (CAR 5.28.z), and was inserted into a pHR’SIN-cPPT-SIEW (pSIEW) lentiviral transfer plasmid upstream of the IRES and eGFP sequences.

The use of gene modification strategies during CIK cell activation and expansion resulted in the WT and genetically modified CIK cells having significantly different expansion rates, particularly between days 3 and 10 of culture. For WT CIK cells, the mean fold change was 25.19 (SD ± 13.673, range 4.6 – 58.10, n = 24), whereas for mock-vector- and ErbB2-CAR-transduced CIK cells, the mean fold changes were 10.27 (SD ± 4.7, range 5.6 – 25.6, n = 24) and 10.23 (SD ± 4.8, range 3.2 – 20.4, n = 24), respectively (Figure 1A). The cell expansion rate was significantly higher among WT CIK cells than among ErbB2-CAR CIK cells (p < 0.0001). However, there were no significant differences between mock-vector and ErbB2-CAR CIK cells with respect to cell expansion rates (p > 0.98).

### Transduction rates and cell surface expression of the ErbB2-CAR

Using lentiviral transduction strategies during CIK cell activation and expansion resulted in mean transduction rates of 23.78% for the ErbB2-CAR vector (SD ± 11.1, range 8.5 – 51.3%, n = 19) and 33.25% for the mock-vector (SD ± 12.4, range 12.1 – 56.4, n = 19) on day 12 of culture (Figure 1B).

On day 12 of culture, the cell surface expression percentage of the ErbB2-specific CAR reached a mean of 78.75% (SD ± 5.7, range 70.6 – 84.6%, n=7) on all ErbB2-CAR vector-transduced cells (Figure 1C).

The molecule that probably plays the most important role in tumor recognition by WT CIK cells is the NKG2D receptor as all CIK cell subsets, even the CD3^+^CD56^-^ T cell subset of CIK cells, exhibit non-MHC-restricted NKG2D receptor-mediated cytotoxic capacity, suggesting these are “*non-classical”* T cells. Detailed flow cytometric analysis of the heterogeneous CIK cell population showed that the ErbB2-CAR fraction mostly contains non-classical CD3^+^CD56^-^ T cells, which account for 92.9% (SD ± 2.65, n = 4) of all transduced cells, while CD3^+^CD56^+^ T-NK and CD3^-^CD56^+^ NK cells only represent 5.47% (SD ± 2.49, n = 4) and 0.45% (SD ± 0.18, n = 4), respectively (data not shown).

### Phenotypic characterization of the WT and ErbB2-CAR CIK cells

#### Analysis of CD3/CD56, CD4/CD8 and T cell receptor (TCR) expression

**Figure 2:**
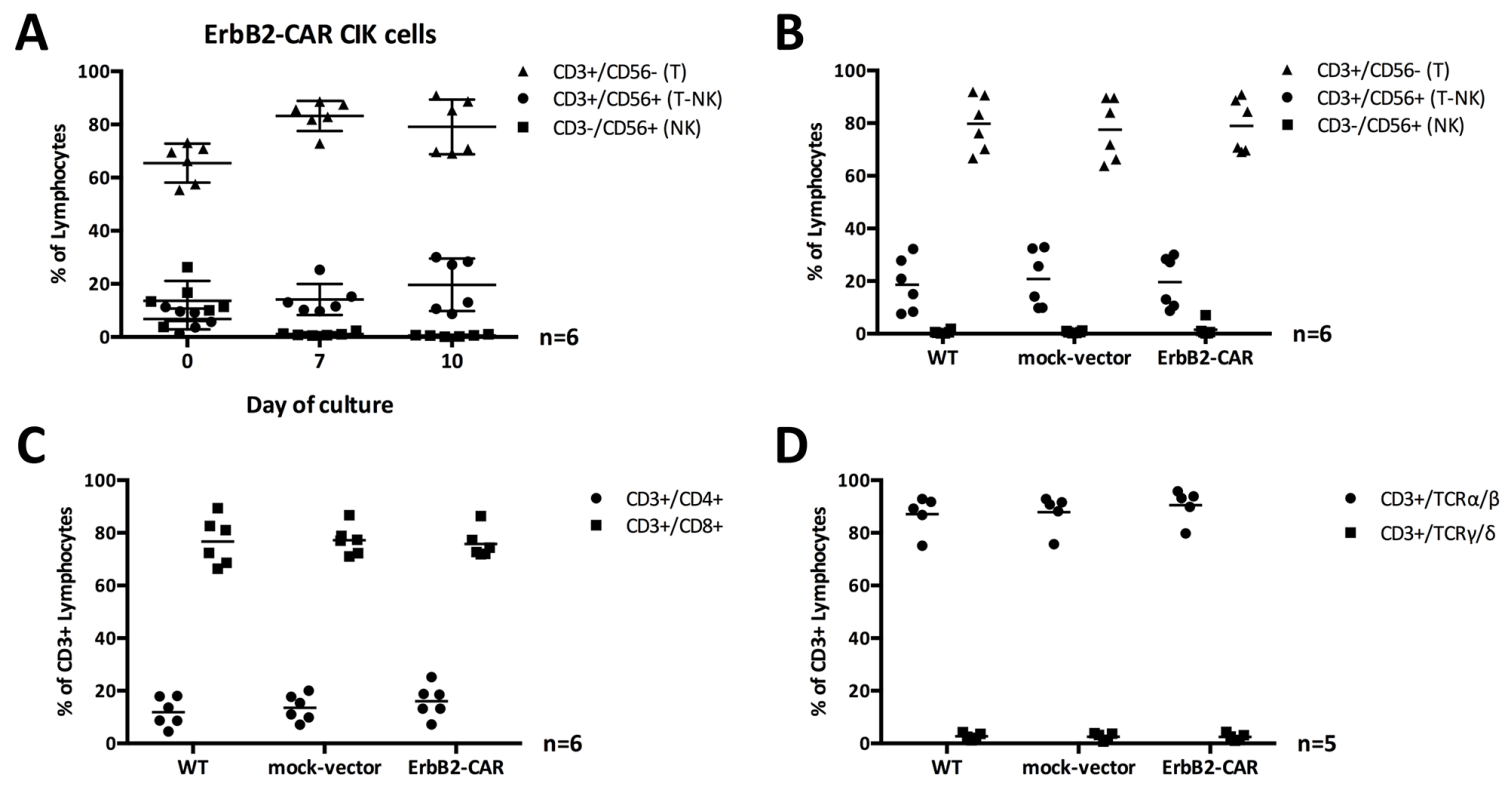
**(A)** CIK cell subpopulations and ErbB2 transduction. T, NK, and T-NK cell subpopulations of ErbB2-CAR CIK cells on days 0, 7, and 10 of culture are shown as percentage of gated lymphocytes. CIK cell subpopulations with CD3^+^ T cell phenotype were preferentially transduced with the ErbB2-CAR. **(B, C, D)** Phenotype of WT and viral vector transduced CIK cells. The percentages of T, NK, and T-NK cells (B) expressing CD4^+^, and CD8^+^ (C), as well as TCRɑ/β, and TCRγ/δ (D) phenotype was compared among WT, mock-vector and ErbB2-CAR CIK cells on day 10 of culture. Generated cells contained predominantly TCRɑ/β T cells with CD4:CD8 ratio of 1:4 - consistent with standard T cells. However, T-NK cells, which expand in the presence of CIK culture conditions and are known as the characteristic effectors among CIK cells were not different in numbers after lentiviral transduction.

To phenotypically compare the WT and genetically modified mock-vector and ErbB2-CAR CIK cells, we performed flow cytometric analyses on days 0, 7 and 10 to 12 of culture. The distribution of CD3^+^CD56^-^ T cells, CD3^+^CD56^+^ T-NK cells and CD3^-^CD56^+^ NK cells was not affected by the genetic modification (n = 6, Figure 2B). In general, the percentage of T-NK cells increased, whereas the NK cells almost disappeared, irrespective of the cell culture conditions. The percentage of T cells increased until day 7 and decreased thereafter, giving rise to T-NK cells in the presence of repetitive stimulation with IL-15 (Figure 2A).

No difference between the sizes of the CD3^+^CD4^+^ T helper and CD3^+^CD8^+^ cytotoxic T cell subpopulations was noted when the genetically modified and WT CIK cell populations were compared (n = 6, Figure 2C). Furthermore, almost all the cells co-expressed TCR-ɑ/β (n = 5, Figure 2D).

#### Analysis of memory phenotype

**Figure 3:**
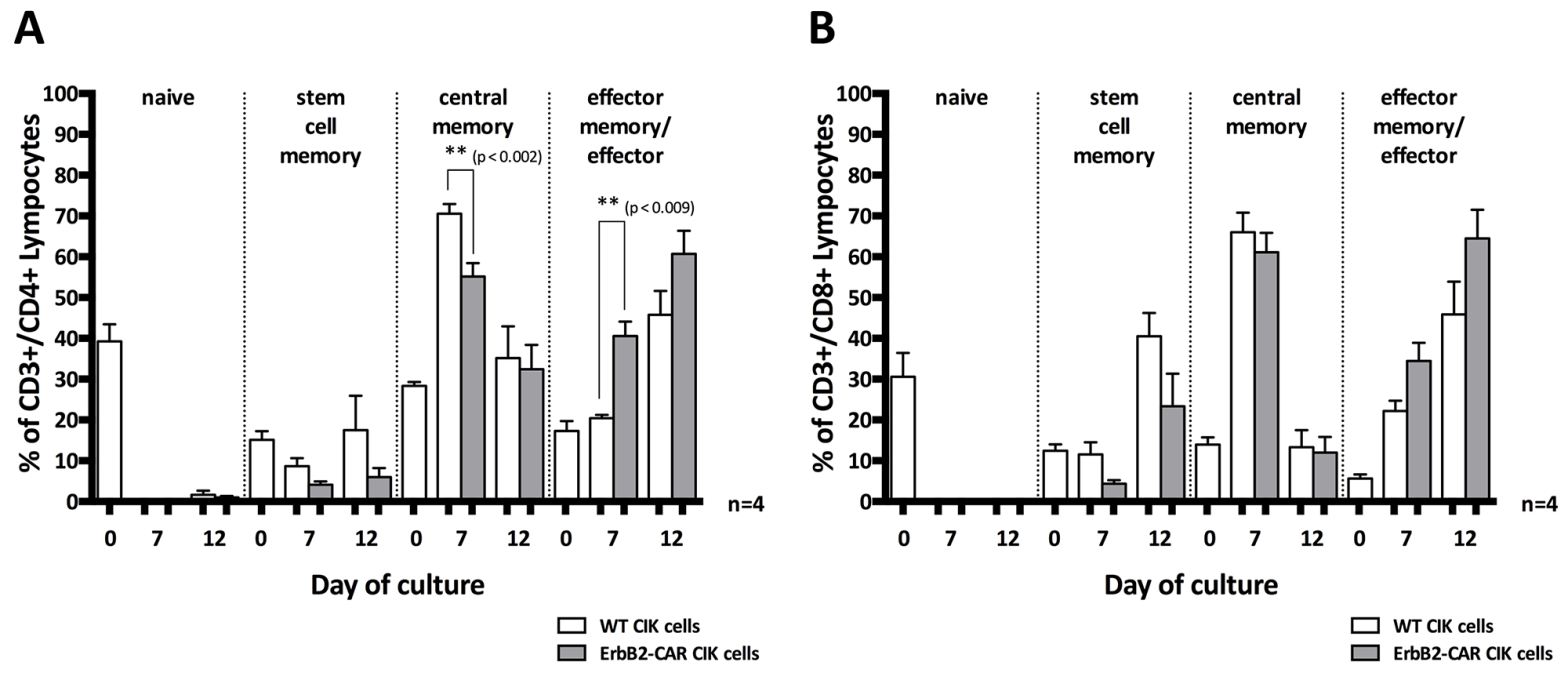
Memory phenotype Changes in the memory phenotypes of WT and ErbB2-CAR CIK expressing CD4^+^ T helper cell phenotype **(A)**, or CD8^+^ cytotoxic T cell phenotype **(B)** are shown in naïve (CD45RO^-^CD62L^+^CD95^-^), effector memory/effector (EM/E, CD45RO^+^/^-^CD62L^-^CD95^+^), central memory (CM, CD45RO^+^CD62L^+^CD95^+^), and stem cell memory (SMC, CD45RO^-^CD62L^+^CD95^+^) subpopulations. On day 7 of culture, significantly higher percentages of differentiated EM/E cells (p < 0.002) and lower percentages of CM cells (p < 0.009) were noted in the ErbB2-CAR CIK cell culture than in the WT CIK cell culture. Regarding the CD8^+^ cytotoxic T lymphocyte subpopulation, we noted that mainly EM/E and SCM cells were present on day 12 of culture.

ErbB2-CAR CIK cells and WT CIK cells showing a T helper phenotype (CD4^+^) displayed phenotypic changes indicating that they had transitioned from naïve (CD45RO^-^CD62L^+^CD95^-^) to mainly effector memory/effector (EM/E, CD45RO^+^/^-^CD62L^-^CD95^+^) and central memory (CM, CD45RO^+^CD62L^+^CD95^+^) cells within 12 days of culture (Figure 3). The stem cell memory (SMC, CD45RO^-^CD62L^+^CD95^+^) subgroup was barely detectable at all time points. However, on day 7 of culture, significantly higher percentages of differentiated EM/E cells (p < 0.002) and lower percentages of CM cells (p < 0.009) were noted in the ErbB2-CAR CIK cell culture than in the WT CIK cell culture (Figure 3A, n = 4).

Regarding the CD8^+^ cytotoxic T lymphocyte (CTL) subpopulation, we noted that mainly EM/E and SCM cells were present on day 12 of culture. Moreover, we found no significant differences between the genetically modified and WT CIK cells with respect to percentages (Figure 3B, n = 4).

### Cell surface expression of ErbB2 and NKG2D ligands on target cells

The molecule that probably plays the most important role in tumor recognition by CIK cells is the NKG2D receptor. NKG2D is a promiscuous receptor that recognizes several counter ligands. These include the MHC-class I-like molecules, MICA and MICB, and members of the ULBP family, named for the ability of some members to bind to the UL-16 protein of cytomegalovirus. The tumor cell lines (MDA-MB-453, THP-1, RH30, RH41, TE671, A204, Renca-lacZ, Renca-lacZ/erbB-2), peripheral blood mononuclear cells (PBMCs) and the primary aRMS VJ cells were analyzed for the expression of the NKG2D ligands ULBP-2/5/6, and MIC A/B, as well as for the expression of ErbB2 (HER2/neu) (Table 1).

**Table 1:** Surface expression of human NKG2D ligands (ULBP-2/5/6, MIC A/B) and human ErbB2 (HER2/neu) on tumor cell lines

Cell line	Cell-type	ULBP-2/5/6	MIC A/B	ErbB2
RH-30	alveolar RMS	+ (47.6%, n = 3)	- (5.0%, n = 3)	+++ (96.7%, n = 5)
RH-41	alveolar RMS	++ (72.1%, n = 3)	- (7.8%, n = 3)	+ (35.1%, n = 5)
VJ [23]	alveolar RMS (primary)	+ (31.7%, n = 3)	- (4.4%, n = 3)	+ (45.7%, n = 5)
A-204	embryonal RMS	++ (88.2%, n = 3)	+ (30.9%, n = 3)	+++ (96.2%, n = 5)
TE-671	embryonal RMS	+++ (90.8%, n = 3)	+ (17.6%, n = 3)	+++ (96.8%, n = 5)
Renca-lacZ [22]	murine renal carcinoma	- (0.1%, n = 3)	- (0.6%, n = 3)	- (0.4%, n = 3)
Renca-lacZ/erbB-2 [22]	w/human ErbB2	- (0.1%, n = 3)	- (1.0%, n = 3)	+++ (94.9%, n = 3)
THP-1	acute monocytic leukemia	++ (79.9%, n = 3)	+++ (91.0%, n = 3)	- (0.1%, n = 5)
MDA-MB-453	breast carcinoma	++ (52.2%, n = 3)	+ (11.8%, n = 3)	++++ (99.5%, n = 5)

- 0 - 10% / + 10 - 50% / ++ > 50 - 90% / +++ > 90 - 99% / ++++ > 99%

(% pos. cells gated on the main population)

### NK cell-restricted cytotoxic capacity

**Figure 4:**
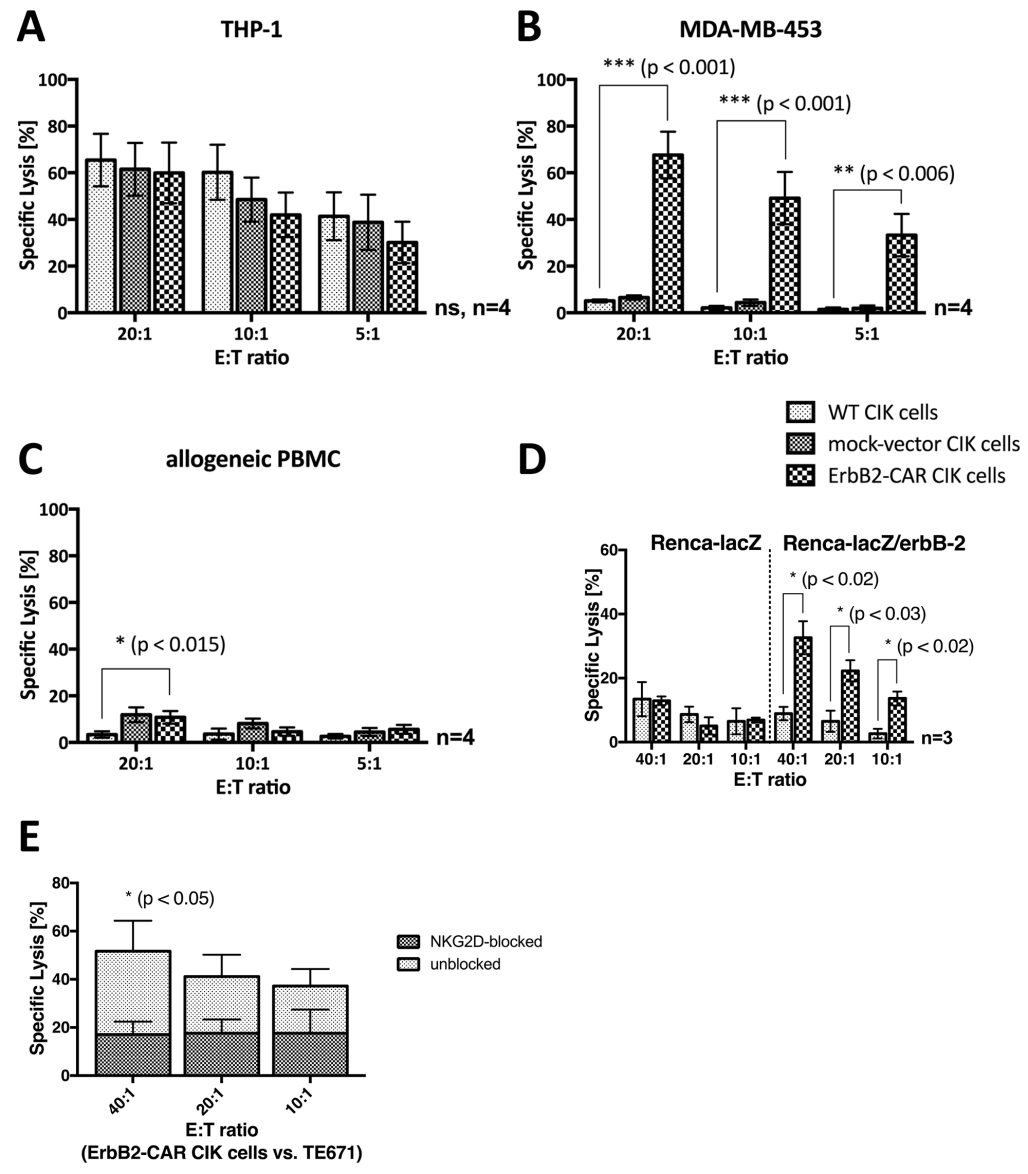
Cytotoxic capacity As a proof of concept, the NKG2D- and ErbB2-mediated cytotoxic capacity of WT and genetically modified (ErbB2-CAR and mock-vector) CIK cells was analyzed by europium release assay against **(A)** ErbB2-negative, NKG2D ligand-positive acute myeloid leukemia target cell line THP-1, **(B)** ErbB2-overexpressing, NKG2D ligand-positive breast carcinoma cell line MDA-MB-453, and **(C)** ErbB2-negative, NKG2D ligand-negative allogeneic PBMCs. The NKG2D-restricted cytotoxicity of CIK cells was not impaired by the genetic modification, shown by the effective cytolysis of ErbB2-negative, NKG2D ligand-positive THP-1 cells by WT, mock- vector, and ErbB2-CAR CIK cells. As expected, the cytotoxic capacity of ErbB2-CAR CIK cells against ErbB2-overexpressing target cells was significantly increased compared with mock-vector and WT CIK cells, and was low against non-malignant ErbB2-negtive, NKG2D ligand-negative target cells. **(D)** Stably transfected mouse renal carcinoma (Renca) cells Renca-lacZ and Renca-lacZ/erbB-2 were used for evaluation of specific ErbB2-targeting by the killer cells. WT CIK cells did not lyse Renca-lacZ and Renca-lacZ/erbB-2 cells. ErbB2-CAR-CIK cells also showed no cytotoxic capacity against Renca-lacZ cells, but displayed highly significant increased cytotoxicity against Renca-lacZ/erbB-2. **(E)** NKG2D mediated cytotoxicity on the cytotoxic capacity of ErbB2-CAR CIK cells against ErbB2 expressing cell line TE671 was evaluated by europium release assay after blocking of the NKG2D receptor on ErbB2-CAR CIK cells. Blocking significantly reduced the efficacy of CAR-modified CIK cells against the tumor target, suggesting that the combination of NKG2D-mediated and ErbB2-specific killing in CAR-CIK cells may reduce the impact of tumor escape mechanisms and increase killing of tumors.

WT CIK cells display a cytotoxicity of a NK cell-restricted nature, and the NKG2D, an activating NK cell receptor, serves as their main effector molecule with respect to target cell recognition. To analyze their capacity for NK cell-restricted cytotoxicity, we co-cultured WT CIK cells and genetically modified CIK cells, which served as effector cells, with the THP-1 cell line, an ErbB2-negative target cell line (Table 1), for 3 hours. We assessed the cytotoxic potential of the cells at effector-to-target cell ratios of 20:1, 10:1 and 5:1 via europium release assay (n=4, Figure 4A), the results of which showed that no statistically significant differences in NK cell-restricted cytotoxicity were present among WT, mock-vector, and ErbB2-CAR CIK cells. However, STS cells in general are relative refractory towards NKG2D-mediated cytotoxicity.

### Cytotoxic capacity against ErbB2-overexpressing target cells

In a proof-of-concept experiment, we assessed the cytotoxic capacity of WT and genetically modified CIK cells against the indicated ErbB2-overexpressing breast carcinoma cell line (MDA-MB-453, Table 1) by europium release assay (Figure 4B). MDA-MB-453 cells were not susceptible to WT CIK cell-mediated cytotoxicity. Therefore, the percentages of WT and mock-vector-transduced CIK cell-specific lysis remained low, even at an effector-to-target cell ratio of 20:1. In contrast, ErbB2-CAR CIK cells displayed a significantly increased specific lysis percentage of 67.6% at an effector-to-target cell ratio of 20:1 (p < 0.001).

### ErbB2-CAR specific cytotoxic capacity

Cytotoxic analysis was performed against the same target with and without ErbB2-expression. Therefore, stably transfected mouse renal carcinoma (Renca) cells Renca-lacZ and Renca-lacZ/erbB-2 (described by Maurer-Gebhard et al. [22]) were used in a 3 hour europium release assay for evaluation of specific ErbB2-targeting by the killer cells. Renca-lacZ control cells do not express human MHC, human ErbB2 or NKG2D ligands, whereas otherwise identical Renca-lacZ/erbB-2 cells express the human ErbB2 target antigen (Table 1). Experiments showed that wildtype CIK cells did not lyse Renca-lacZ and Renca-lacZ/erbB-2 cells. ErbB2-CAR-CIK cells also showed no cytotoxic capacity against Renca-lacZ cells, but displayed highly significant increased cytotoxicity against Renca-lacZ/erbB-2 (n=3, Figure 4D). These data demonstrate specific cytolysis of target cells via the ErbB2-CAR.

### NKG2D-mediated target cell recognition and killing of ErbB2-CAR CIK cells

To address the question if the cytotoxic potential of ErbB2-CAR CIK cells is also mediated through both, the NKG2D receptor as well as the ErbB2-CAR, blocking experiments were performed against ErbB2 expressing TE671 cells. NKG2D blocking significantly reduced the efficacy of CAR-modified CIK cells against the tumor target (n=3, Figure 4E). Hence, with the whole ErbB2-CAR CIK cell population, NKG2D- and ErbB2-mediated cytotoxicity are applied together.

### Cytotoxic capacity against allogeneic PBMCs

We analyzed the cytotoxic activity of WT and genetically modified CIK cells against allogeneic PBMCs (Figure 4C). WT CIK cells showed no cytotoxic activity against allogeneic PBMCs, even at an effector-to-target cell ratio of 20:1 (mean 3.4%), while mock-vector and ErbB2-CAR CIK cells exhibited slightly higher activity levels (mean 10.8% for ErbB2-CAR CIK cells, p < 0.015). No differences in cytotoxic capacity were noted between mock-vector-transduced and ErbB2-CAR-transduced CIK cells (p > 0.79 at 20:1 E:T).

### Cytotoxic potential against RMS cell lines and primary RMS cells

We assessed the capacity of CAR-engineered CIK cells to recognize and kill ErbB2-expressing RMS target cells (Table 1) by europium-release assay on day 12 of culture after co-incubation for 3 hours and by brightfield imaging cytometry after co-incubation for 16 hours.

**Figure 5:**
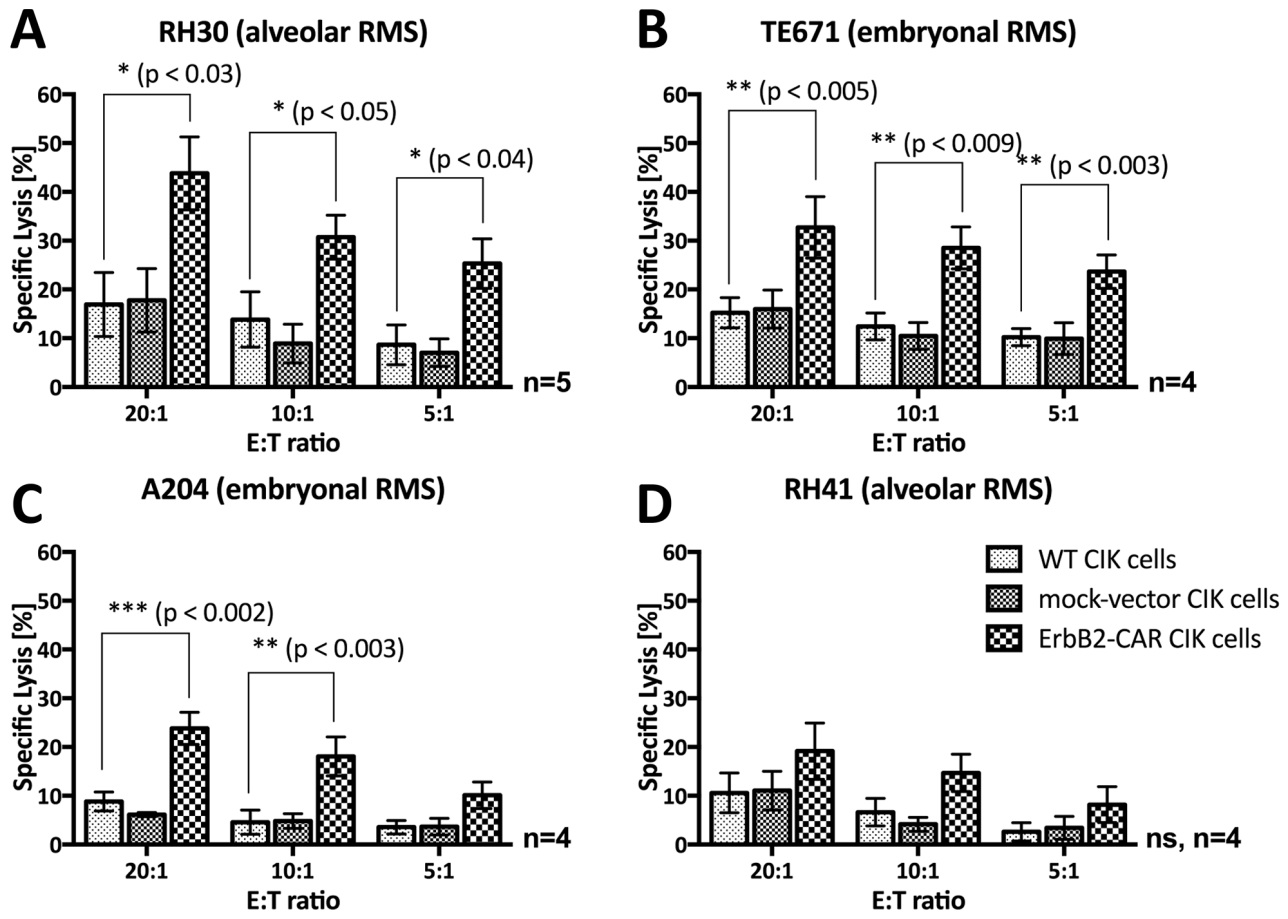
Short-term (3-hour) anti-tumor potential Cytotoxic analysis of WT and genetically modified (ErbB2-CAR and mock-vector) CIK cells against ErbB2-expressing **(A)** RH30 (aRMS) **(B)** TE671 (embryonal RMS), **(C)** A204 (embryonal RMS) and **(D)** RH41 (aRMS) cells using a 3-hour europium release assay are shown. ErbB2-CAR transduction significantly increased the anti-tumor potential of CIK cells against the RH30, TE671, and A204 RMS cell lines within 3-hour co-culture of effector and target cells. Hereby, ErbB2-CAR CIK cytotoxicity correlated with ErbB2 antigen expression levels.

In a 3-hour assay, ErbB2-CAR CIK cells showed potent and increased *in vitro* cytolytic activity against aRMS RH30 cells (Figure 5A) and embryonal RMS TE671 (Figure 5B) and A204 cells (Figure 5C) compared with WT CIK cells at effector-to-target cell ratios of 20:1 and 10:1. The increase in ErbB2-CAR CIK cell cytotoxicity against RH30 and TE671 cells was also significant at a 5:1 effector-to-target cell ratio. ErbB2-CAR CIK cells also displayed increased cytotoxicity against RH41 cells (Figure 5D) in the 3-hour assay, although this increase was not significant. We noted no significant difference in cytotoxic potential between mock-vector-transduced and WT CIK cells.

**Figure 6:**
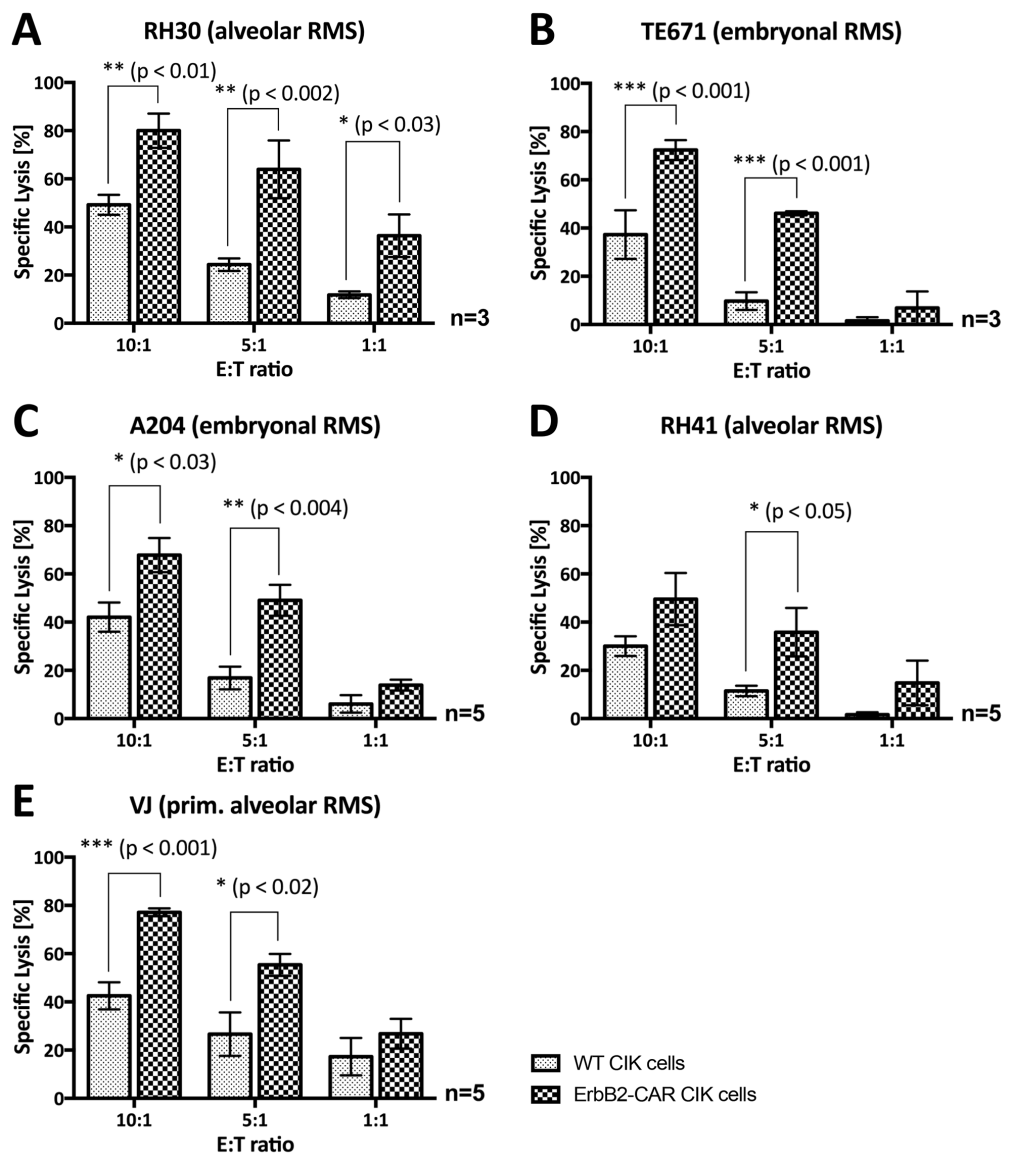
Overnight (16-hour) anti-tumor potential Analysis of the cytotoxic capacity of WT and genetically modified (ErbB2-CAR) CIK effector cells against ErbB2-expressing **(A)** RH30 (aRMS) **(B)** TE671 (embryonal RMS), **(C)** A204 (embryonal RMS), **(D)** RH41 (aRMS), and **(E)** primary aRMS cells using brightfield imaging cytometry after 16-hour co-incubation of effector and target cells is illustrated. ErbB2-CAR transduction increased the anti-tumor potential of CIK cells against RMS cell lines, as well as against refractory aRMS cell line RH41 (Figure 3D) and primary RMS cells at very low effector-to-target cell ratios of 10:1, 5:1 and 1:1.

After the effector and target cells were co-cultured for 16 hours, ErbB2-CAR-engineered CIK cells displayed significantly increased cytotoxicity against all the RMS cell lines, even at low effector-to-target cell ratios of 5:1 or 1:1 (Figure 6).

The cytotoxic capacity of WT, mock-vector-transduced and ErbB2-CAR CIK cells against primary aRMS VJ cells [23] with ErbB2 surface expression (Table 1) was assessed by bright field imaging cytometry using a Celigo cell cytometer (Figure 6E). In contrast to the established cell lines, which originated from the RMS metastases of heavily pretreated patients, the VJ cells were generated from the primary tumor of an untreated patient. The cytotoxicity results showed that WT CIK cell activity levels were significantly lower than ErbB2-CAR CIK cell activity levels. At an effector-to-target cell ratio of 10:1, WT CIK cells displayed a mean specific lysis percentage of 42.5%, and ErbB2-CAR CIK cells displayed a mean specific lysis percentage of 77.1% (p < 0.001, n = 5).

### Cytotoxic capacity against RMS monolayers and RMS spheroids

Several tumor models were established for a better mimic of the clinical situation. To analyze the cytotoxicity capacity of WT and ErbB2-CAR CIK cells against attached growing target cells, we cultured RMS (RH30) cells in flat-bottom culture plates. WT CIK cells recognized RMS cell monolayers, but were neither able to sufficiently separate the adherent growing RMS cell populations from the culture bottoms nor could lyse the cells. In contrast, ErbB2-CAR CIK cells specifically recognized and rapidly killed their tumor targets within a few hours (Supplementary Video 1).

**Figure 7:**
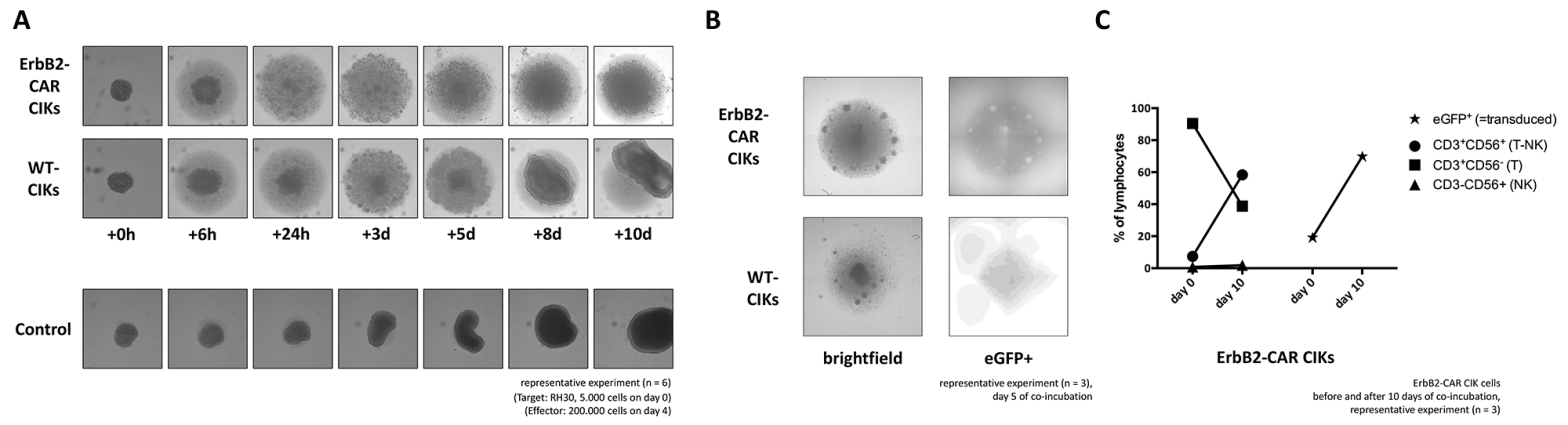
Cytolysis of tumor spheroids ErbB2-positive tumor spheroids comprising of 5,000 RH30 cells (aRMS) were co-incubated with 200,000 WT or ErbB2-CAR CIK cells. **(A)** The cultures were imaged after 6 hours and every 24 hours thereafter. **(B)** Using the fluorescence imaging microscope of a Celigo cell cytometer, clusters of eGFP-positive ErB2-CAR CIK cells were detectable on day 5 of co-culture, suggesting proliferation of ErB2-CAR CIK cells in the presence of tumor targets. **(C)** Flow cytometric analysis of effector cells before and at the end of co-culture showed an increase of eGFP-positive ErbB2-CAR CIK cells, mainly expressing CD3^+^CD56^-^ T cells, and in particular CD3^+^CD56^+^ T-NK cells, known as characteristic CIK cell effectors.

In contrast to tumor monolayers, tumor spheroid models mimic the three-dimensionality of tumors. Tumor spheroids comprising RH30 aRMS cells were generated and co-incubated with effector cells, as described below. WT CIK cells could infiltrate the tumor spheroids but failed to destroy them and were therefore overgrown by recurrent tumor cells at the end of the co-incubation period. In contrast, ErbB2-CAR CIK cells infiltrated the tumor spheroids within the first 24 hours of co-culture. Furthermore, after three days of co-culture (mean 3 days; range 2 – 4 days, n = 3), no tumor cells remained in the presence of ErbB2-CAR CIK cells (Figure 7A). Accordingly, no ErbB2-positive target cells were identified by flow cytometry at the end of co-culture (data not shown). Further flow cytometric analysis showed that among both WT and ErbB2-CAR CIK cells, the percentage of CD3^+^CD56^+^ T-NK cells increased (Figure 7C, only ErB2-CAR CIK cells shown), and among ErbB2-CAR CIK cells, the percentage of eGFP (=transduced)-positive CD3^+^ cells increased from 19.23% to 69.84% during co-culture with the corresponding target cells. Fluorescence microscopy analyses confirmed eGFP-positive CAR CIK cell clustering in the presence of the corresponding tumor antigens (Figure 7B).

## DISCUSSION

The prognosis of patients with advanced-stage, relapsed, or refractory STS treated by standard and experimental means, including high-dose chemotherapy with autologous stem cell rescue and allogeneic stem cell transplantation as forms of cell therapy, remains poor [24, 25]. Likewise, our recent clinical trial data showed that non-specific (WT) CIK cell therapy following allogeneic stem cell transplantation did not improve outcome. Considering autologous CIK cell therapy in patients with advanced-stage, relapsed, or refractory STS, we asked whether additional tumor-specific CIK cells - engineered with CARs - may help clear tumors and prevent tumor recurrence without providing additional toxicity. Here, we intended to determine whether STS cells express targetable tumor antigens that can be used to redirect WT CIK cells modified with the corresponding CAR and whether this CAR-CIK cell approach may improve cytotoxic potential against STS expressing the targetable antigen in several tumor models without providing additional toxicity compared with WT CIK cells. Furthermore, expansion rates and phenotypes were compared with WT CIK cells.

T cells genetically modified to express CARs have shown great promise with respect to the treatment of CD19-positive hematological malignancies [9-12]. Even malignancies featuring minor subsets of cells with CD19-positivity and exhibiting very low levels of antigen expression have responded to CD19-targeted CAR T cell therapy [26]. However, the clinical experience regarding the use of tumor antigen-specific T cell therapy in the treatment of solid tumors is limited [27, 28].

GD2, IL-13Ra2, B7H3, and ErbB2 have been identified as potential targetable tumor antigens on solid tumors, including bone sarcomas and Ewing’s family tumors [29]. ErbB2 induces malignant transformation and tumorigenesis in NIH 3T3 fibroblasts, indicating that ErbB2 functions as an oncogene that is also correlated with a poor prognosis in malignancy, e.g., in sarcoma [30, 31].

Using FACS analysis, we noted that ErbB2 was expressed at low levels on all tested STS cell lines and on an established primary rhabdomyosarcoma cell line. Therefore, ErbB2 is also expressed on other than bone sarcomas and Ewing’s family tumors, such as soft tissue sarcomas, including high-risk, relapsed and refractory RMS with alveolar histopathological characteristics, albeit at low levels, making this tumor target clinically relevant. In contrast, immunohistochemistry analysis failed to detect ErbB2 expression on primary tumor sections (data not shown), suggesting that immunohistochemistry may not be sufficiently sensitive to detect low levels of ErbB2 expression on patient samples [32].

Verneris et al. showed that even low levels of ErbB2 expression could be used to redirect CIK cells to tumor targets using a CD3-ErbB2-bispecific antibody, which efficiently induces cytolysis of tumor targets both *in vitro* and *in vivo* [33]. These findings are consistent with those of studies involving genetic modified T cells that used CARs and showed that cells exhibiting low levels of ErbB2 expression can also be targeted by ErbB2-CAR T cells [29, 34].

The CAR used by Sung Hee Yoon at al. in a study in which CIK cells were redirected against ErBb2 tumor targets contained a different anti-ErbB2 antibody fragment but was also joined to the intracellular portions of CD28 and CD3ζ, as was the case for the CAR construct used in our study [21]. In 2010, Morgan et al. reported a third-generation ErbB2-CAR [35]. The CAR constructs used in this study employed the scFv fragments of different ErbB2-monoclonal antibodies (trastuzumab vs FRP5), which bind to distinct epitopes [36, 37]. Additionally, the trastuzumab-based third-generation CAR contained a CD28.CD137.ζ endodomain, which is known for its antigen-independent T cell activation [38]. Ahmed et al. recently demonstrated the safety and activity of ErbB2-CAR T cell therapy in 17 patients with sarcomas in a phase I/II clinical trial [13] using the same second-generation CAR that was used in our study.

Sung Hee Yoon et al. showed that CIK cells may represent an alternative effector cell source for CAR therapies compared with standard T cell approaches [16-18, 20, 21]. CIK cells are *in vitro*-expanded immune effector cells exhibiting T and NK phenotypes. Interestingly, CIK cells in part do recognize target cells through the T cell receptor (TCR) but do not require the presence of major histocompatibility complex (MHC) molecules on target cells. The anti-tumor activity of CIK cells mainly relies on the engagement of NKG2D by NKG2D ligands on tumor cells, and on perforin-mediated pathways. Hence, even the CD3^+^CD56^-^ T cell subset of CIK cells exhibits non-MHC-restricted NKG2D receptor-mediated cytotoxic capacity, suggesting these “*non-classical”* T cells contrast with (CAR-) T cells generated from PBMCs described by Steven Rosenberg et al. [11, 39, 40].

Transduction methods and expansion protocols for transduced cells are important points. In our study, we used CIK cells and gene transfer technology relying on vectors derived from retro- or lentiviruses, like most CAR T cell studies. Transduction was performed after cytokine activation with interferon gamma, interleukin (IL)-2, low-dose anti-CD3 antibody, and IL-15 on days 4 - 5 of culture. This is consistent with gene transfer and RNA electroporation technologies used in previous reported CIK cell approaches. Moreover, Magnani et al. recently reported the nucleofection of PBMCs using an improved Sleeping Beauty transposon platform [41]. Interestingly, in our setting mostly CIK cells with a CD3^+^ T cell phenotype were lentivirally transduced with the ErbB2-CAR, showing donor dependent transduction rates ranging from 8.5 – 51.3%. The transduction efficiency may suggest intrinsic problems of CIK cells, but transduction was feasible in all cases. Furthermore, similar data and transduction rates were also reported for CAR T cells in previous studies [42, 43].

While T cells and T-NK cells were transduced efficiently with the lentiviral CAR vector, the small NK-cell subpopulation was not effectively genetically modified. This is in agreement with the literature showing that transduction of pure and short-lived NK cells is difficult and predominantly restricted to NK cell lines [18, 44]. This is not considered problematic, since the NK cell population represents a minor and rather insignificant fraction of CIK cells, suggesting no relevant cytotoxic potential provided by this population.

Transduced cells were further expanded in the presence of IL-15 resulting in a mean ErbB2-CAR expression percentage of 78.75% at the end of culture. Expansion data confirmed adequate expansion in CAR expressing CIK cells, yet diminished expansion in the presence of viral vector, but with higher percentages of differentiated effector memory/effector cells compared to cells cultured without viral vector. Those cells are highly active against tumor targets, which might allow lower doses of ErbB2-CAR CIK cells still assuring robust ErbB2-specific cytotoxic potential. Similar findings have been seen by many groups, likely related to non-specific toxicity of the vector itself.

Nevertheless, generation of high numbers of CAR-CIK cells is a very important issue for further preclinical and clinical *in vivo* studies. Therefore, transduction might be performed earlier like shown by Magnani et al. [41]. In addition, CIK-cytokine-cocktails might be changed or adapted to CAR-T cell generation (IL-2/IL-7/IL-15, [45]). Also, particles or beads loaded with CD2, CD3, and CD28 antibodies may be used for activation and expansion. In addition, the culture period might be prolonged to increase ErbB2-CAR-CIK cell recovery. To exclude problems related to the virus itself, the SBS transposon platform might be considered for future use [41].

Detailed phenotypic analysis of WT and ErbB2-CAR CIK cells showed that CD3^+^CD56^-^ T cell, CD3^+^CD56^+^ T-NK cell and CD3^-^CD56^+^ NK cell levels were not affected by CAR-engineering. Especially, the T-NK cell phenotype, known as terminally differentiated immune effector cells arising from T cell progenitors and representing the main effectors among WT CIK cells [1, 46], was retained in ErbB2-CAR CIK cells. However, after 10-12 days of *in vitro* culture T-NK cells are a very rare population among IL-15-activated CIK cells, which is an evident contrast to 21 days of IL-2 expansion.

Generated cells contained predominantly TCR ɑ/β T cells with CD4:CD8 ratio of 1:4, consistent with standard T cell populations. Analysis of the memory phenotype showed that significantly higher percentages of differentiated EM/E cells and lower percentages of CM cells were present among ErbB2-CAR CIK cells than among WT CIK cells, suggesting that ErbB2-CAR CIK cells are more terminally differentiated and therefore may be more active than WT CIK cells. Interestingly, SCM cells, which were present at very low levels on day 7 of culture, seemed to evolve from the CM subgroup rather than from the naïve T cell subgroup. Replenishment of ErbB2-CAR CIK cells, as well as WT CIK cells, may be the result of the two cell lines evolving from these persisting SCM cells.

Preclinical *in vivo* as well as clinical studies underlined the importance of the whole CIK cell population and the originating T cell progenitors among CIK cells [1, 47-49]. To prevent reduced functionality previously observed for sorted CIK cell subpopulations, we used unselected CIK cells with and without CAR-expression for cytotoxicity analysis. Our cytotoxicity studies showed that NKG2D-restricted target cell recognition and killing were retained after CIK cell genetic modification, as ErbB2-negative THP-1 cells expressing NKG2D ligands were also targeted and effectively killed by ErbB2-CAR CIK cells, suggesting that ErbB2-CAR CIK cells are also active by NKG2D. Specific cytolysis of target cells via the ErbB2-CAR was demonstrated using mouse renal carcinoma (Renca) cells with our without human ErbB2 expression [22], which do not express human MHC or NKG2D ligands. ErbB2-CAR-CIK cells showed no cytotoxic capacity against Renca-lacZ cells, but displayed highly significant increased cytotoxicity against Renca-lacZ/erbB-2 cells. Third-party PBMCs lacking NKG2D ligands were only moderately killed by WT and ErbB2-CAR CIK cells. Although, cytotoxicity was low this may represent a concern with need to be further analyzed. However, considering the use of ErbB2-CIK cells in the autologous setting, as have most CAR T cell studies, CAR-engineered T cells have proven to be surprisingly safe, despite treatment of now many hundreds of patients with many hundreds of patients’ years’ follow-up. In contrast, ErbB2-CAR CIK cells demonstrated significantly increased cytolytic potential against ErbB2-expressing target cells, including primary tumor cells. Notably, ErbB2-CAR CIK cells were also active in tumors with low ErbB2 expression. This was not the case for WT CIK cells. Furthermore, cytotoxic capacity of WT and ErbB2-CAR CIK cells against attached growing RMS cells showed that WT CIK cells recognized RMS cells, but were neither able to sufficiently separate the adherent growing RMS cell populations from the culture bottoms, nor were they able lyse these cells. In contrast, ErbB2-CAR CIK cells specifically recognized, separated and rapidly killed their tumor targets. Three-dimensional tumor spheroid models, as intermediates between monolayer culture and *in vivo* tumors, comprising of RMS cells were not infiltrated and lysed by WT CIK cells, but by ErbB2-CAR CIK cells which in addition expanded due to tumor recognition. Additional proliferative potential in this case may have been provided by the costimulatory domain of the second-generation CAR. Altogether, the superior recognition, infiltrating, proliferating and killing capacities of ErbB2-CAR CIK cells compared to WT CIK cells was proven in 16-hour cytotoxicity analyses, in tumor monolayers and tumor spheroids, conditions which best mimic *in vivo*-like models in which tumor cells outnumber immune effector cells. Within these co-cultures, we identified expanding ErbB2-CAR-expressing T cells and T-NK cells as the main effectors, suggesting that ErbB2-CAR CIK cell-mediated cytotoxicity is both an ErbB2-CAR- and a NKG2D-restricted phenomenon. Furthermore, blocking NKG2D significantly reduced the efficacy of CAR-modified CIK cells against ErbB2-expressing tumor targets, suggesting that the combination of NKG2D-mediated and ErbB2-specific killing in CAR-CIKs may reduce the impact of tumor escape mechanisms and increase killing of tumors with a relatively low and heterogeneous expression of ErbB2, such as most STS tumors.

Clinical studies with CAR T cell strategies, showed that T cells exhibit the capability to home to tumor sites, as well as to proliferate, persist, and release cytokines upon encountering tumors. In comparison, these capabilities were minimal among WT CIK cells showing limited anti-tumor activity in preclinical analysis [50-53]. However, CAR engineering may comprise synergism or even additive effects of CAR- and NKG2D-mediated killing provided by CIK cells. Preclinical *in vivo* and clinical data on this issue are desirable.

Taken together, our results indicate that ErbB2-CAR redirection of CIK cells improves the cytotoxicity of immune effector cells against ErbB2-positive tumor targets compared to WT CIK cells, implying that this therapy may represent a step forward in the treatment of resistant, relapsed and advanced STS.

## MATERIALS AND METHODS

### Primary target cells and target cell lines

THP-1, an AML M4 subtype cell line, was obtained as previously described [46]. The human breast carcinoma cell line MDA-MB-453 was obtained from ATCC (Manassas, VA, USA). The aRMS cell lines RH30 and RH41, as well as the embryonal RMS cell lines TE671 and A204, were purchased from DSMZ (Deutsche Sammlung von Mikroorganismen und Zellkulturen GmbH, Braunschweig, Germany). Primary aRMS VJ cells were kindly provided by the research group of Prof. Dr. Fulda (J.W. Goethe University Frankfurt, Institute for Experimental Cancer Research in Pediatrics, Frankfurt, Germany) [23]. These primary tumor cells were generated from a patient diagnosed with histologically confirmed aRMS and cultured in standard DMEM supplemented with fetal calf serum, glutamine, penicillin and streptomycin as previously described [23]. Stably transfected mouse renal carcinoma cells Renca-lacZ and Renca-lacZ/erbB-2 [22] were generated by genetically modifying the murine renal carcinoma cell line Renca with the human EbB2 target antigen. The cells were cultured in standard RPMI supplemented with fetal calf serum, glutamine, penicillin, streptomycin, Zeocin (Renca-lacZ and Renca-lacZ/erbB-2) and G418 (Renca-lacZ/erbB-2 only). The commercially available cell lines were cultured according to their manufacturers’ instructions.

### Generation of wild-type (WT) CIK cells

WT IL-15-activated CIK cells were generated from PBMCs (Figure 1D) of healthy volunteers, as previously described [46], after written informed consent was provided by the volunteers, and the study was approved by the Ethics Review Board of the Medical Faculty of the University Hospital Frankfurt/Main, Germany (Geschäfts-Nr. 413/15). To establish comparable culture conditions for the WT CIK cells and mock-vector and ErbB2-CAR-engineered CIK cells (described below), we cultured WT CIK cells on 6-well plates from day 4 or 5 to day 7 of culture. Cell density was maintained approximately 5 x 10^5^ cells/2 mL. On day 7 of culture, the cells were transferred to culture flasks. Cell density was maintained at approximately 1 x 10^6^ cells/mL, and 50 ng/mL IL-15 was added every 3-4 days until day 10 of culture. On day 10 to 12 of culture, the cells were harvested and used for further analysis.

### The ErbB2-specific lentiviral CAR vector pS-5.28.z-IEW

The lentiviral CAR vector pS-5.28.z-IEW, which encodes an ErbB2-specific second-generation CAR, was described previously [54]. The codon-optimized CAR sequence consists of an IgG heavy-chain signal peptide, an ErbB2-specific antibody fragment scFv (FRP5) and a modified CD8α hinge region, as well as CD28 transmembrane and intracellular domains and a CD3ζ intracellular domain (CAR 5.28.z), and was inserted into a pHR’SIN-cPPT-SIEW (pSIEW) [55] lentiviral transfer plasmid upstream of the IRES and eGFP sequences. eGFP was used as a fluorescence marker gene (Figure 1E). pSIEW (without the CAR-sequence) was used as mock-vector control.

### Generation of viral particles

VSV-G pseudotyped lentiviral vector particles were produced using the lentiviral transfer plasmid (including either the CAR-sequence, pS-5.28.z-IEW or, in the case of the mock-vector control, the pSIEW sequence), as well as the packing and envelope plasmids pCMVΔR8.91 and pMD2.G as described previously [56].

### Engineering of CIK cells

CIK cell CAR engineering was performed on day 4 or 5 of CIK cell culture, 24 hours after stimulation with IL-15 (Figure 1D). The CIK cells were seeded onto standard 6-well plates, adjusted to a cell density of 5 x 10^5^ cells per well and covered with 1 mL of medium. To generate mock-vector or ErbB2-CAR CIK cells, we added 1 mL of the corresponding vector supernatant (mock-vector or ErbB2-CAR) containing 16 μg of hexadimethrine bromide (polybrene; Sigma Aldrich, St. Louis, MO, USA; 8 μg/ml) to the plates, which were subsequently centrifuged at 1800 g at 32°C for 60 minutes. On day 7 of culture, the cell suspensions were removed from the 6-well plates, washed twice and cultured at a density of 1 × 10^6^ cells/mL in fresh medium supplemented with 50 ng/mL IL-15. Cell density was maintained at approximately 1 x 10^6^ cells/mL, and 50 ng/mL IL-15 was added every 3-4 days until day 10 of culture. On day 10 to 12 of culture, the cells were harvested and used for further analysis.

### Cell surface staining of the target and effector cells

Aliquots of tumor and CIK cells were analyzed for the expression of various cell surface markers. The CIK cells were analyzed on days 0, 7 and 10 to 12 of culture, and the tumor cells were analyzed on their day of use as target cells as part of a cytotoxicity analysis. The monoclonal antibodies (MABs) used herein were conjugated with fluorescein isothiocyanate (FITC), peridinin chlorophyll (PerCP), phycoerythrin (PE), phycoerythrin-cyanin 7 (PE/Cy7), allophycocyanin (APC), allophycocyanin-cyanin 7 (APC/Cy7) or Pacific Blue™ (PB) and directed against CD3 (PerCP), CD8 (APC, APC/Cy7), CD4 (PE), CD56 (PE/Cy7), TCRα/β (PE), TCRγ/δ (APC), CD62L (PB), CD95 (PE/Cy7), CD45RO (APC), CD45 (PE/Cy7), CD340 (ErbB2/HER2-neu; PE), ULBP-2/5/6 (PE) and MIC A/B (PE). All the antibodies were obtained from BioLegend (San Diego, CA, USA) unless otherwise specified. To detect the cell surface expression of ErbB2-CARs, we labeled the CIK cells with an ErbB2 fusion protein as the primary reagent (ErbB2-IgG-Fc chimera, Sino Biological Inc., Beijing, P.R. China) after unspecific Fc-receptor blocking using TruStain FcX (Fc-Receptor Blocking Solution, BioLegend, San Diego, CA, USA). A secondary anti-IgG-Fc monoclonal antibody conjugated with APC was used to detect the primary ErbB2-IgG-Fc chimera.

Gates were set on viable lymphocytes or tumor cells, and the data for 5 - 30 x 10^4^ events were acquired using a BD FACSCanto II flow cytometer (BD Biosciences, San Jose, CA, USA) using FACSDiva software (Version 6.1.3, BD Biosciences). Analyses were performed using FlowJo X software (Version 10.1, Tree Star Inc., Ashland, OR, USA). All multicolor flow cytometry assays with 2 or more colors were adjusted for spectral overlaps. Isotype-matched fluorchrome-conjungated antibodies were used as controls.

### Europium-release assay for analysis of short-term cytotoxicity

We used the europium-release assay to perform an analysis of short-term (3-hour) *in vitro* cytotoxicity, as previously described [5]. Blocking of NKG2D was achieved by adding 30 μg of human NKG2D (CD314) antibody (R&D Systems, Minneapolis, MN, USA) per 4 x 10^6^ CIK cells. After incubation for 4 hours at standard culture conditions the effector cells were used in an otherwise regular europium-release assay.

### Analysis of long-term cytotoxicity by automated imaging using a Celigo cell cytometer

To analyze long-term cytotoxicity (16-hour) we plated adherent growing target cells in a flat-bottom 6-well plate (Corning Incorporated, Corning, NY, USA) with an absolute density of 5 x 10^5^ cells per well. After a period of 4 to 6 hours, we removed the supernatants and non-adherent cells by gently washing the culture plates twice with 2 mL of phosphate buffered saline (PBS) before adding one milliliter of medium to each well. For cell counting, we used a Celigo cell cytometer (Nexcelom Bioscience LLC., Lawrence, MA, USA), which features an automated cell analysis system that uses direct brightfield imaging. Images were acquired and analyzed using direct cell counting applications to identify and count individual cells. After determining the initial target cell counts, we added WT, mock-vector or ErbB2-CAR CIK cells to the wells in duplicate or triplicate at effector-to-target cell ratios of 10:1, 5:1 and 1:1. The cultures were maintained in 2 mL of medium, and target cell cultures without effector cells were used as negative controls. After a culture period of 16 hours, we removed the floating dead cells by withdrawing the supernatants and gently washing the culture plates twice with 2 mL of PBS. One milliliter of PBS was subsequently added to each well, and the number of remaining adherent target cells was counted using a Celigo cell cytometer. The percentage of specific cytolysis was calculated as follows: 1 – mean number of remaining target cells / mean number of remaining cells of the corresponding negative controls.

### Co-culture in the presence of tumor spheroids

Three-dimensional tumor spheroids were generated with a single spheroid per well [57] and automatically imaged using a Celigo cell cytometer (colony counting embryoid body application). To generate the tumor spheroids, we used ultra-low attachment (ULA) 96-well round-bottom plates (Corning Incorporated, Corning, NY, USA) without additional coating. After adding 200 μL of target cell suspension (cell density, 25 x 10^3^ target cells/mL) to each well, we centrifuged the culture plates with an absolute number of 5000 target cells per well at 1000 g for 10 minutes. Half of the culture medium was replaced every 3 to 4 days. On day 4 of tumor spheroid culture, we added 200,000 WT or ErbB2-CAR CIK cells to each well. The culture conditions were visualized 6 hours later and every 24 hours thereafter. On day 10 of co-culture, the tumor spheroids and effector cells were harvested and used for further analysis.

### Video analysis of cytotoxic capacity

To analyze the cytotoxic capacity of WT and ErbB2-CAR CIK cells, we plated attached growing target cells in a flat-bottom 6-well plate with an absolute density of 5 x 10^5^ cells per well. After a culture period of 4 to 6 hours, we removed the supernatants and non-adherent cells by gently washing the culture plates twice with 2 mL of PBS. WT or ErbB2-CAR CIK cells were subsequently added to the plates at an effector-to-target cell ratio of 2:1. The cultures were performed in 2 mL medium, and the co-cultures were video-analyzed for a period of 16 - 18 hours.

### Statistical analysis

Differences between values were calculated using the two-tailed Student’s t-test. A p-value < 0.05 was considered significant. Statistical analyses were performed using GraphPad Prism software (Version 6e, GraphPad Software, La Jolla, CA). The results are presented as mean values ± standard errors of the mean (Figures) and mean values ± standard deviations (data in the Results section).

## SUPPLEMENTARY MATERIALS VIDEO




