# Supplementary material for: Generation and characterization of ErbB2-CAR-engineered cytokine-induced killer cells for the treatment of high-risk soft tissue sarcoma in children
Source: Oncotarget. 2017 Aug 2;8(39):66137–53. doi: 10.18632/oncotarget.19821 (PMC5630399; doi:10.18632/oncotarget.19821)
Supplement: Supplementary file 1 [file oncotarget-08-66137-s001.pdf]

Generation and characterization of ErbB2-CAR-engineered cytokine-induced killer cells for the treatment of high-risk soft tissue sarcomas in children

SUPPLEMENTARY MATERIALS

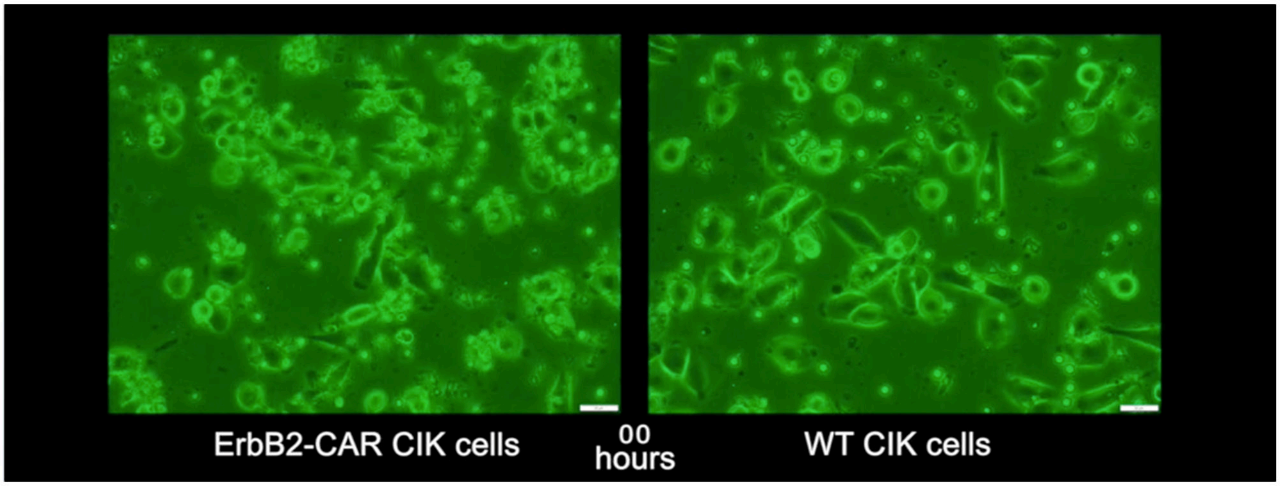

Supplementary Video 1: Time-lapse (21h) video (200x magnification) of co-incubation of WT (right) or ErbB2-CAR CIK cells (left) and RMS (RH30) at an effector-to-target cell ratio of 2:1 is shown. Time of co-incubation (hours) is indicated in the middle. Cytotoxicity capacity of WT and ErbB2-CAR CIK cells against attached growing RMS cells showed that WT CIK cells recognized RMS cells, but were neither able to sufficiently separate the adherent growing RMS cell populations from the culture bottoms, nor were they able lyse these cells. In contrast, ErbB2-CAR CIK cells specifically recognized, separated and killed their tumor targets, and expanded due to target cell recognition.
